# Would Reducing Children’s Exposure to Food Advertising Prevent Unhealthy Weight Gain?

**DOI:** 10.1007/s13679-025-00648-6

**Published:** 2025-06-21

**Authors:** E. Boyland

**Affiliations:** Department of Psychology, University of Liverpool, Liverpool, L69 7ZA UK

**Keywords:** Food, Advertising, Marketing, Digital, Children, Weight gain, Obesity, Regulation

## Abstract

**Purpose of Review:**

To provide and consider evidence for the role of food advertising in childhood obesity development and reflect on the efficacy of current policy interventions to restrict children’s food advertising exposure.

**Recent Findings:**

Children are exposed to extensive advertising for unhealthy foods, particularly online. Visually appealing techniques and salient themes are employed by food marketers to capture children’s attention and provoke engagement and emotionally driven responding. Food advertising exposure adversely affects children’s food behaviors, and the relationship with obesity meets established criteria for causality. Theoretical models proposing likely pathways underpinning observed behavioral effects have gathered empirical support. Implemented restrictive food advertising policies have achieved reductions in exposure, persuasive power, and purchasing of unhealthy foods, though no country has yet implemented comprehensive regulations across all forms of food advertising and marketing.

**Summary:**

Actions to reduce children’s food advertising exposure, and the persuasive power of that exposure, are an important part of an effective approach to preventing childhood weight gain and obesity and reducing health inequalities. Tackling digital food marketing is challenging but essential to public health efforts given its ubiquitous and influential presence in children’s lives.

## Introduction

Childhood obesity has reached epidemic proportions and is a pressing global public health challenge that requires bold leadership to address [1]. It is well documented that children from socioeconomically disadvantaged and ethnic minority background face greater health risks from obesity [2] and that public health interventions that intervene at the macro system level should be prioritized [3]. The World Health Organization has urged governments to address childhood obesity and reduce health inequalities by implementing a range of population level interventions, including food and beverage (hereafter: food) advertising restrictions [4]. In the context of these calls to action, this article seeks to provide a contemporary overview of the extent and nature of children’s food advertising exposure, the methodological challenges inherent in quantifying the personalized commercial content delivered via digital devices, emerging tools supporting progressive digital monitoring research, the current state of evidence of food advertising impact on eating behaviors and body weight in children, the psychological, physiological and sociocultural factors that may explain these effects, and what we know of the effectiveness of implemented policies in limiting food advertising exposure to reduce childhood obesity. Unhealthy food advertising and the need to support healthy diets and child body weights transcends national boundaries, therefore this overview of the latest evidence has clear global relevance.

## Children’s Contemporary Food Advertising Exposure and its Measurement

There is extensive food advertising in the media and settings frequented by children and it is predominantly for unhealthy products [5]. Similar to observed trends in obesity prevalence, there is evidence of socioeconomic patterning in food advertising exposure, such that children from ethnic minority and disadvantaged households and communities are disproportionately exposed [6]. Most monitoring research, until recently, has been focused on television advertising as a traditionally major source of commercial exposure for children. The largest such study to date, a global collaboration across twenty-two countries spanning the Asia Pacific region, Africa, Central and South America, Europe and North America, found that, on average, 23% of television commercials on channels popular with children were for food or beverage products and there were four times more advertisements for unhealthy than healthy items [7].

However, in recent years, the internet has become increasingly more prominent in children’s lives and media use patterns. With greater leisure time online invariably comes greater exposure to digital marketing, most often on portable devices such as smartphones and tablets [8]. The World Health Organization (WHO) define digital food marketing as promotional content for food via digital devices, but also acknowledge the unique characteristics that set digital apart from other advertising forms – particularly the personalization and data-driven, targeted delivery based on contextual and behavioral profiling [9]. This personalization of digital advertising presents notable ethical, legal, and methodological challenges for researchers seeking to monitor children’s exposure to inform appropriate regulatory actions [10]. In recognition of this, WHO Europe produced the ‘CLICK’ monitoring framework as an overarching guide to digital marketing investigation [11], supported by a package of protocols providing practical step-by-step guidance for researchers seeking to monitor digital marketing across a number of digital platforms including video-sharing sites, social media, and game streaming platforms [12].

These, and other emerging digital advertising monitoring approaches (for a comprehensive review, see [13]), have recently been applied in several countries, and the resulting studies provide meaningful, novel insights into the digital advertising landscape and the extent of children’s contemporary digital advertising exposure. Content analyses reporting on the landscape of digital advertising identify what can be considered children’s ‘*potential* or *inferred* exposure’. In one study, researchers assessed 926 posts by top selling food and beverage products and brands across the most popular social media platforms used by children and adolescents in Mexico (Facebook, Instagram, and YouTube), finding that 93% of the food promoted on posts that appealed to children and adolescents had an unhealthy nutritional profile [14]. In Brazil, food advertising was found to be present in 12.9% of popular videos on YouTube’s most-watched channels with content aimed at children, and more than 90% of commercials were for ultra-processed products [15]. A study in Canada reported that YouTube had the highest rate of food marketing by social media influencers, followed by Tik Tok and Instagram, with fast food and sugar sweetened beverages the most promoted food categories, and overall, 83% of products/brands were assessed to be unhealthy [16]. Further, Potvin Kent and colleagues used commercial data to determine that marketing by the 40 food brands that frequently target youth in Canada reached billions of social media users across four platforms in 2020, with the most reach achieved by fast food restaurants and sugar sweetened beverage brands [16].

To study children’s *actual* exposures to digital advertising, researchers have recently begun using screen capture approaches to allow real time quantification of the volume of commercial content delivered to the digital devices used by children. In the largest such study to date, 347 child and adolescent participants in Mexico recorded their digital device screen for 45 min while they were online [17]. A majority (69.5%) were exposed to digital food marketing during this period, at a median rate of 2.7 exposures per hour (47.3 per week). The most frequently marketed foods were ready-made foods and > 90% were unhealthy based on their nutrient profile. In Australia, 95 adolescents (13–17 years) recorded their mobile device screen for two weekdays and one weekend day whenever they visited relevant online platforms [18]. Analysis of these videos found that the participants saw a median of 17.4 food promotions each hour that they were on the internet. Extrapolated based on usual time online, this would mean typical exposure to 168.4 digital food promotions per week, of which 99.5% were deemed unhealthy according to WHO nutritional criteria [19]. Given the resource intensive nature of such studies, in the context of concerns about a lack of advertising monitoring data for low- and middle-income countries [8, 20], it has since been identified that evaluating a subsample of just 30% of children’s usual screen time is sufficient to provide reliable estimates of their habitual digital food advertising exposures [21]. As technology and capabilities develop, it is likely that further efficiencies may be achieved using artificial intelligence (AI) based approaches to automate some aspects of the monitoring process [13, 22].

## Persuasive Techniques and Children’s Engagement with Digital Food Advertising

The persuasive power of advertising results, at least in part, from the use of specific techniques within commercial content that make the message persuasive and compelling, though the strength of the persuasive effect may vary across the different creative elements and different audiences [23]. Consistent with this, the use of themes in Instagram food marketing by the largest fast food company in the world has been shown to vary between high income, upper middle income and lower middle income countries [24]. Specifically, a greater proportion of the social media posts in lower-middle-income countries featured child-targeted themes (22%) and price promotions and free giveaways (40%) compared with high-income countries (12% and 14% respectively).

Similar variation in message characteristics has been found for soda brand social media marketing across countries of higher and lower Socio-demographic Index [25]. There also appear to be individual differences in the techniques that children find appealing; in a Canadian study a group of children aged 6–12 years showed low agreement as to the advertising components (e.g., text, logos, cartoons, color, fun themes) in digital food marketing content that best predicted child appeal [26]. The visual and artistic features of the social media platforms themselves may also factor into food advertising appeal, as a study found that adolescents rated food ads more positively with Instagram features than without [27].

Recent content analyses, particularly of commercial content on digital platforms popular with youth, have documented the most frequently occurring persuasive techniques and themes used to promote unhealthy foods. In a study of the brand pages for 20 of the Philippines’ most popular food products and brands on three leading digital media platforms (Facebook, YouTube, Instagram), fun was most central to the advertising of food (between 18–24% of social media posts and videos), followed by taste (11–19% of posts), and enjoyment/satisfaction (13–18% of posts) [28]. Analysis of food marketing identified in the screen captures of Mexican children’s devices (as described in the previous section) found that the most frequently used product-related strategies were showing images of products, packages, and consumption/purchase incentives, while in terms of persuasive strategies, brand characters were most frequently appearing, followed by celebrities and competitions or contests [17]. Differences in the content of posts deemed to be targeting and not targeting adolescents have also been observed, whereby graphic design elements (bright colors, fonts, drawings, animated effects) and language or expressions commonly used by adolescents or young adults were key indicators of advertising targeting adolescent audiences [29].

Concerns about the use of tactics such as ‘health-washing’ (creating an aura of health around unhealthy foods [28]) and ‘green-washing’ (using marketing claims that do not accurately reflect companies’ environmental activities, exaggerate these activities, omit information on their real environmental footprint, or make vague or false statements on this issue [30]) in food advertising have also gained prominence in recent years. For example, physical activity regularly features in social media advertisements appealing to children, with some messaging overtly implying that consumption of the promoted products was associated with energy and sporting success [28]. Similarly, food advertising on gaming platforms, largely for products such as energy drinks, snacks, and food delivery firms and apps [31, 32], has been noted to often use messaging that suggests their products can improve gaming performance and stamina [8]. In a content analysis of unhealthy food marketing on Instagram in Uruguay, it was found that more than half of all posts featured at least one cue (visual or textual) conveying health-related associations such as healthy product composition, healthy lifestyle, or health benefits of consumption [33]. Such tactics have been suggested to be particularly manipulative as exposure to this marketing contributes to the creation of implicit beliefs and associations about products and brands that may not be accurate (e.g., that an unhealthy product is associated with fitness and health) and thus could undermine informed consumer choice as well as public health actions aiming to reduce consumption of unhealthy foods [5, 28, 33]. With respect to food marketing tactics, the Covid-19 pandemic was an interesting case study in both the misappropriation of health and societal causes by food brands for competitive advantage and also the incredible agility of large transnational corporations to rapidly adjust marketing tactics in light of changing consumer behaviour, for example by shifting focus to support for social distancing and emphasising empathy [34, 35].

A study exploring food marketing on TikTok identified the most common marketing strategies to be branding (87% of videos), images of products (85%), engagement (31%) and appearance of celebrities or influencers (25%) [36]. The term ‘engagement’ in this context is usually used to refer to digital marketing in particular and the actions of “liking”, “sharing” or “following food and beverage brands on social media” [23]. Brands often actively seek and promote brand engagement through their marketing activities (e.g., via so-called ‘hashtag challenges’ [36]), this is perhaps unsurprising given that engagement has been shown to be enhance children’s behavioral responses to marketing exposure [37]. Engagement also drives amplification of exposures associated with positive sentiments [36] that can persist even beyond the duration of the brand’s advertising campaign [38]. Adolescents, in particular, appear to have high engagement with food brands online. A significantly greater percentage of US adolescents (13–17 years) were found to follow food brand accounts (9.2%) than other accounts (1.2%) on Twitter (now ‘X’), this was not the case for Instagram though here adolescents were more likely to follow sugary drink brands (7.9%) than the low-calorie equivalents (4.3%) [39]. In another study of US adolescents (also 13–17 years), a large majority (70%) reported engaging with food brands on social media, with 35% regularly engaging with more than five different food brands [40].

Unhealthy food marketing appears highly memorable for young people. In semi-structured group interviews with adolescents (11–18 years) in Uruguay, researchers found participants were readily able to recall being exposed to digital food marketing, particularly for fast food and food-ordering apps, and could point to images, colors, music, portion size, product novelty, price promotions and celebrity endorsement as the most memorable features of this marketing [41]. Further, in a study using fictitious Facebook profile feeds to explore Irish adolescents’ responses to social media advertising posts, adolescents recalled and recognized a greater number of unhealthy food brands (compared with healthy food brands or non-food advertising), viewed these posts for longer, and were more likely to wish to share unhealthy posts as well as rating peers more positively when they had unhealthy posts in their feeds [42].

## Impact of Food Advertising on Children’s Eating Behaviors and Body Weight

Evidence of the relationship between exposure to food advertising and eating and related food behaviors in children and young people has grown substantially in recent years. A cross-sectional study involving over a thousand Belgian adolescents found significant associations between self-reported exposure to food marketing on social media and greater preferences for and intake of unhealthy foods [43]. Similarly, self-reported exposure to food or beverage advertising on social media within the past week was associated with greater intake of unhealthy beverages in a large (n = 8708) sample of Australian adolescents [44]. One study demonstrated longitudinal effects of social media influencer marketing in a sample of 8–12 year old children, finding that their self-reported frequency of exposure to this marketing influenced consumption of unhealthy beverages two years later [45].

These effects have also been examined experimentally. A study in Uruguay randomly assigned 433 adolescents (12–18 years) to be exposed to a clothing commercial, a burger commercial, a salad commercial, or no commercial [46]. The effect of this exposure on subsequent choices from a mock menu was moderated by both sex and burger consumption frequency (i.e., the biggest influence on choice was seen in males who regularly consume burgers). Published reviews have noted the occurrence of gender-based differences in food advertising impact across multiple studies [47], as well as the existence of a moderating effect of body mass index, such that the greatest magnitude of behavioral response is seen in children with overweight or obesity [48].

Numerous recent systematic reviews and meta-analyses have synthesized relevant experimental and observational studies of food advertising impact. One found that the presence of a promotional character (compared with no character) on unhealthy food packaging resulted in children reporting significantly greater taste preference for unhealthy products [49]. A series of meta-analyses conducted as part of the development process for the updated WHO global guidelines [50] on food marketing restrictions in 2023 found significant associations between food marketing exposure (via digital media, television, or food packaging) and increased immediate intake in children, as well as altered food choices and preferences in favor of unhealthy, marketed foods [51]. Increased food intake effects have also been identified specifically for exposure to digital marketing via social media and advergaming [52] and digital games or influencers [53]. The mean difference in children’s energy intake between exposure to food advertising and control exposures has been identified as 57.7kcal [48], which is a substantial proportion of the estimated energy gap required for the development of weight gain in children (110–165kcal/day) [54].

Our detailed understanding of the relationship between food advertising and childhood obesity is limited by a lack of longitudinal evidence and inclusion of weight related outcomes in relevant studies [51]. This gap is perhaps unsurprising, given the substantial methodological difficulties inherent in any attempt to quantify the relative contribution of food advertising to a multifactorial disease that is known to be largely driven by small, cumulative increases in energy intake at the individual level [55]. These difficulties include the perpetual and integrated exposure of children to food advertising as they go about their daily lives, such that isolating its effect experimentally with the required degree of ecological validity would be expensive and hugely challenging [56]. However, self-reported food commercial exposure has been shown to be indirectly associated with children’s body mass index in some [57] but not all [58] cross-sectional studies. Importantly, the relationship between exposure to food advertising and obesity in children meets all criteria typically used in epidemiology to demonstrate the existence of causal relationships – namely: strength of evidence; consistency of findings; specificity; temporality; biological gradient; plausibility; coherence; experimental evidence; and analogy [56].

## Potential Pathways Linking Food Advertising Exposure and Childhood Weight Gain

**Fig. 1:**
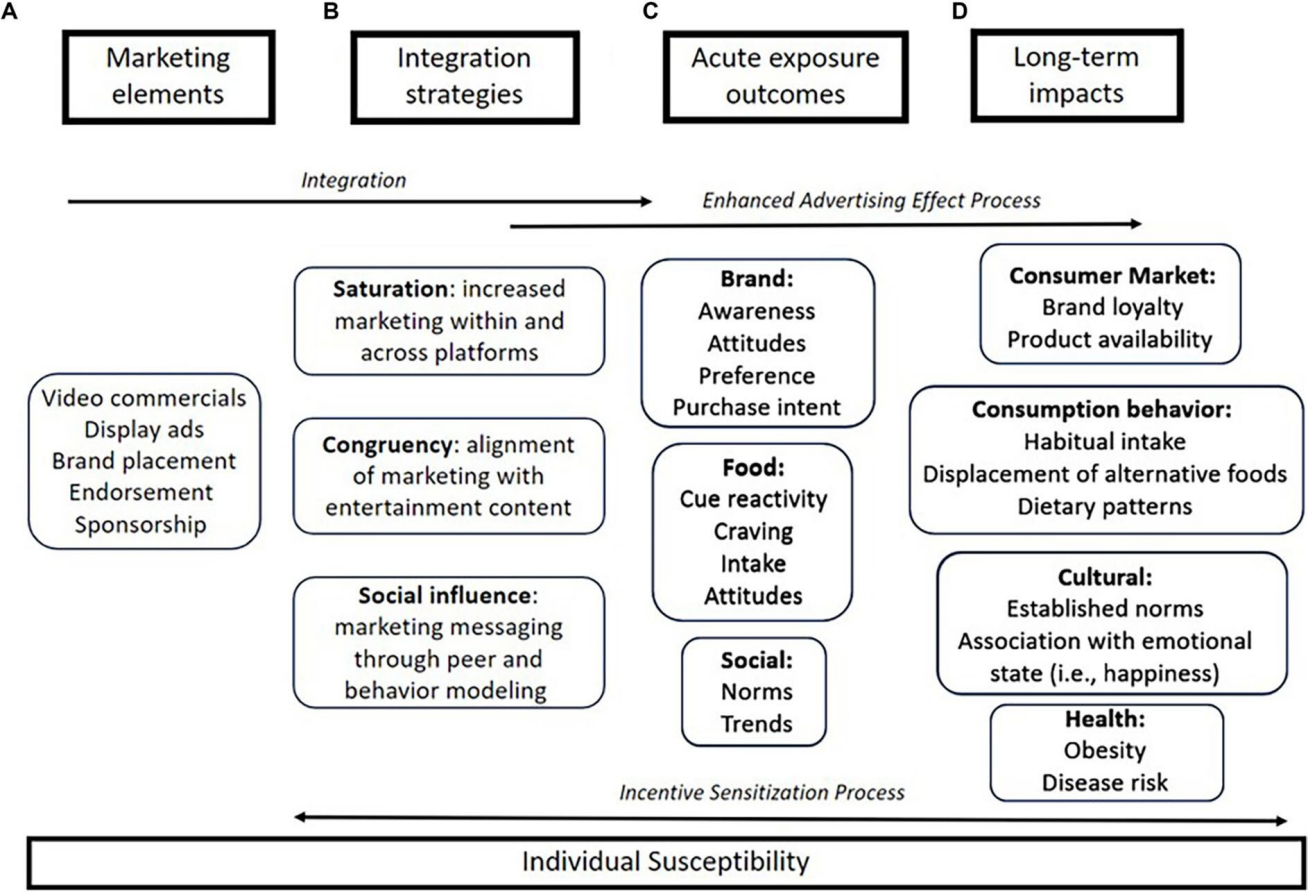
The food and beverage cues in digital marketing (FBCDM) model (originally published in [61]). Copyright 2024 Maksi, Keller, Dardis, Vecchi, Freeman, Evans, Boyland and Masterson

A growing number of mechanistic and theoretical studies now speak to the potential pathways underpinning these observed behavioral effects and their relation to weight gain in childhood. There has been notable progress in the development and validation of psychological models that can be applied to understand how food marketing affects children since the influential Food Marketing Defense Model in 2009 [59]. For example, the Reactivity to Embedded Cues in Advertising Model (REFCAM) describes how marketing influences and reinforces the consumption of unhealthy foods by inducing physiological and psychological processes to motivate viewers to obtain and consume the advertised food, with this consumption, in turn, increasing susceptibility to future advertising exposures [60]. Building on REFCAM, the food and beverage cues in digital marketing (FBCDM) model (Fig. 1) incorporates key marketing elements and integration strategies that are common to digital media and are thought to enhance and amplify the behavioral sensitization process and advertising effects [61]. Consistent with evidence that food marketing affects norms and sociocultural values underpinning foods [62] alongside immediate consumption and related behaviors [51], the FBCDM hypothesizes that repeated food marketing exposure has significant longer term consequences in terms of consumption behaviors, culture, health as well as consumer markets [61]. A recent cross-sectional study has lent further support to the importance of considering norms in the pathway of food advertising effects, finding that descriptive norms mediated the positive association between exposure to unhealthy food on social media and unhealthy food consumption in Flemish adolescents (11–19 years) [43].

The FBCDM also recognizes the unique capability of digital media to harness the salience of emotional cues in food advertising [61], a notion supported by experts in an interview study who identified that emotional engagement and the attachment of symbolic meanings to foods were integral to the processing of social media food marketing [63]. In support of this, a recent meta-analysis of whole brain neuroimaging studies highlighted that food advertising exposure elicits consistent activation of brain regions relating to emotional processing, as well as visual processing, attention, sensorimotor activity in children and adults [64]. Further evidence of the likely physiological mechanisms underpinning food advertising effects emanates from the identification of differences in child and adolescent brain response in reward- and attention-related regions between unhealthy and healthy digital food images and varying portion sizes of food [65], as well as differential effects on children’s neural responding across advertising mediums (dynamic versus static) [66].

Ten years ago, Kelly and colleagues identified that evidence (largely based on studies of the impact of television food advertising) supported a logical sequence of effects, including cognitive, attitudinal, affective, and behavioral outcomes, linking food advertising exposure with body weight and health [67]. Since then, the pathways of the model have been tested in several cross-sectional studies and have been found to have empirical support. In a study of 2,260 UK children (7- 11 years), commercial television exposure was found to be directly associated with children’s purchase requests, purchasing, and consumption of unhealthy foods as well as indirectly associated with children’s body mass index via purchasing and consumption through purchase requests [57]. Similarly, recall of unhealthy food marketing on videogame livestreaming platforms was found to be significantly associated with purchase and consumption of marketed food categories in a sample of UK adolescents (13–18 years), mediated by attitudes to unhealthy foods [58]. A further study with 400 Australian children (10–16 years) found that children’s commercial screen media use was significantly and positively correlated with their attitudes towards brands and their brand purchasing behaviors (including own purchases and purchase requests to parents) [68].

## Effectiveness of Restrictive Food Advertising Policies for the Prevention of Childhood Weight Gain

While no country has yet implemented comprehensive regulations effectively restricting unhealthy food advertising in all its forms [69, 70], many governments globally are starting to implement legislative actions in this area [71]. Notably, in the most recent and comprehensive global synthesis of evidence evaluating the effectiveness of implemented food marketing policies, no studies reported on body weight or obesity outcomes [69]. However, some policies were associated with reduced exposure of children to unhealthy food advertising, reduced use of powerful persuasive techniques by advertisers, lower food advertising spend, and reduced purchasing of unhealthy foods [69]. Policy evaluations consistently find that mandatory Government-led approaches are more effective than voluntary self-regulation initiatives by the food industry [69, 72]. Importantly, even when self-regulatory initiatives apply stricter nutrition criteria, evidence shows that companies do not deliver on their promises to advertise healthier products to children [73]. Other key policy design components that appear to affect the effectiveness of policies in meeting regulatory goals include the age range of children sought for protection, how marketing to that demographic is defined, which media and forms of marketing are restricted, and how the specific foods to be restricted are classified [69].

Oft cited as one of the most comprehensive approaches taken globally [74], Chile’s food labelling and advertising law (implemented 2016) restricted, among other things, unhealthy food marketing considered to be directed to or intended for children younger than 14 years [75]. Post-implementation, there is evidence of significant reductions in the prevalence of unhealthy food advertisements around television programs intended for children [76], household purchasing of unhealthy foods [77, 78], and children’s consumption of such foods [79]. The later phase added time-based, as well as child-based, restrictions on television food advertising and was more effective in reducing children’s exposure [80].

Using the effect sizes drawn from evidence of food advertising’s impact on energy intake (as described earlier), modelling studies have been conducted to simulate the potential effects of policies to restrict food advertising for the purposes of childhood obesity prevention. An Australian study estimated that legislation to restrict unhealthy food advertising before 9.30pm on television would reduce children’s energy intake by an average of 115kJ per day and body mass index by an average of 0.352kg/m^2^ [81]. Importantly, the benefits were predicted to be greater in the most disadvantaged children. Similarly, a UK study tested the notion that all unhealthy food advertising before 9pm was withdrawn and found that children’s daily caloric intake would reduce by 9.1kcal which would reduce the number of children with overweight (including obesity) by 3.6%, with reductions approximately two fold greater in the least affluent social grade relative to the most affluent [82]. This study was timely, given that the UK Government has enacted a progressive law to restrict unhealthy food product advertising (within specified categories of relevance to children’s diets) not only on television before 9pm but also all paid-for marketing of those products online [83]. After several delays, this legislation is due to be formally implemented in January 2026. Researchers have devised a concept map to illustrate how reactions of various stakeholders to these regulations could either reinforce or undermine the impact of these policies on the commercial food system and public health (including obesity), as well as to inform the design of appropriate evaluations [84]. The methodological challenges inherent in quantifying the potential impact of the online component of such policies were highlighted in an important paper by Tatlow-Golden and Parker [85]. Their examination of the premise, assumptions, and analysis undertaken for the Government’s own impact assessment found substantial (tenfold, if not more) underestimation of children’s exposure to digital food marketing which, in turn, severely limited the predicted benefits of the policy as part of efforts to tackle childhood obesity.

## Evaluating the Effectiveness of Policies Restricting Food Marketing

Best practice design principles indicate that governments should take the lead in establishing systems for monitoring policy compliance and evaluating the impacts of implemented policies, with frameworks for these actions ideally developed during the policy design phase and included in the legislation and associated government documents [13, 74]. The monitoring and evaluation framework should specify the priority media and settings to be assessed, the frequency of data collection and key indicators [13]. Both the World Health Organization [12] and the INFORMAS network [13] have published standardized monitoring protocols which can guide governments in designing such frameworks in such a way as to facilitate comparisons across time and place. For example, evaluating the impact of policies to reduce marketing exposure and power requires detailed assessments across media and settings, including data acquired prior to implementation for baseline metrics and repeating the same approach post-implementation for comparison. System-level concept maps can support the design of complexity-informed evaluations, that go beyond assuming singular cause-effect pathways to consider how complex systems adapt and flex when interventions, such as a new restrictive policy, are introduced [84]. Attributing changes in any outcome to the policy versus underlying secular trends can be challenging due to the absence of a counterfactual or control group in natural experiments, but interrupted time series designs can mitigate for some of these challenges [13, 69]. These designs have been shown to be useful in recent evaluations of local food marketing policies [86] and similar national public health regulations [87].

As noted earlier in this paper, there are substantial measurement challenges inherent in understanding children’s digital food marketing exposure (and therefore also understanding whether and how this has changed following implementation of a restrictive policy). However, wherever possible (with due acknowledgement of the resource implications), actual exposure should be measured as it has been established that industry data does not capture all digital advertising spending, and spend data does not meaningfully translate into information on ‘reach’ (exposure) [85].

Evaluations should ideally be carried out by an independent institution, with a clear mandate from government which allocates the necessary resources required for benchmarking and ongoing monitoring of the impact and any unintended consequences [74]. For example, restrictive policies seeking to protect young children from exposure to unhealthy food marketing may inadvertently increase exposures for older children and adolescents [69, 88]. If policy elements are adjusted post-implementation, or there is a stepwise implementation process, then monitoring should be extended to ensure that the data captured allows for informed consideration of whether an extension to the scope of the regulations is warranted [74].

## Conclusions

Unhealthy food advertising is a pervasive adverse influence on children’s food behaviors, including choice, preference, and food intake. Food marketers use a range of themes, techniques and appeals in advertising content to ensure it is engaging and persuasive, often tailoring these to attract youth audiences. The growth of digital media has amplified food advertising exposure and engagement for children, who readily recall the specific features of commercial content they have seen online. Recent psychological models incorporate digital features and consider the pathways of effect, including physiological, sociocultural, and behavioral factors, between food advertising exposure and changes in body weight. It appears increasingly likely, based on the latest neuroimaging data, that the demonstrated impacts of food advertising on diet result from emotionally driven behavioral responding. Meaningfully isolating and quantifying the specific contribution of food advertising to child weight gain is hugely challenging, if not impossible, though cross-sectional relationships with body weight have been observed and evidence meets established criteria for causality. Robust restrictive policies provide opportunities for effective population level intervention to tackle childhood obesity, with effective implemented policies being associated with reductions in advertising exposure, power, and purchasing of unhealthy foods. Monitoring and evaluation should be considered during policy design and carried out by independent institutions in line with best practice principles and standardized protocols. There is progress with legislative action proposed or undertaken in several countries and moves to regulate the digital food environment will be critical to promoting good dietary health and healthy weight in children.

## Key References

- World Health Organization. Policies to protect children from the harmful impact of food marketing: WHO guideline. Geneva: World Health Organization; 2023. Licence: CC BY-NC-SA 3.0 IGO. Accessible from https://www.who.int/publications/i/item/9789240075412. 2023.
  - Evidence-based recommendations and implementation considerations for policies to protect children from the harmful impact of food marketing.
- Kelly, B., et al., Contemporary Approaches for Monitoring Food Marketing to Children to Progress Policy Actions. Curr. Nutr. Rep., 2023. **12**(1): p. 14–25.
  - Monitoring approaches for food advertising, including in online media, to progress policy actions, availability of protocols, training and support for researchers in low- and middle-income countries.
- Boyland, E., et al., Association of Food and Nonalcoholic Beverage Marketing With Children and Adolescents’ Eating Behaviors and Health: A Systematic Review and Meta-analysis. JAMA Ped., 2022. **176**(7): p. e221037-e221037.
  - Comprehensive synthesis of global evidence that food advertising exposure significantly affects food choice, preference, and intake in children.
- Sing, F., et al., Designing legislative responses to restrict children’s exposure to unhealthy food and non-alcoholic beverage marketing: a case study analysis of Chile, Canada and the United Kingdom. Globalization Health, 2022. **18**(1): p. 72.
  - Synthesis and analysis of the technical elements of food advertising laws to support governments in developing effective restrictions.

## Data Availability

No datasets were generated or analysed during the current study.
